# Early ophthalmological tumour signs and diagnostic interval in children with brain tumours

**DOI:** 10.1038/s41433-025-03837-8

**Published:** 2025-05-17

**Authors:** Jacob Christiansen, René Mathiasen, Steffen Heegaard, Sia Kjeldsen, Kjeld Schmiegelow, Volkert Siersma, Sarah Linea von Holstein

**Affiliations:** 1 Department of Ophthalmology, Copenhagen University Hospital Rigshospitalet-Glostrup, Copenhagen, Denmark; 2 Department of Paediatrics and Adolescent Medicine, Copenhagen University Hospital Rigshospitalet, Copenhagen, Denmark; 3 Department of Clinical Medicine, Faculty of Health Sciences, University of Copenhagen, Copenhagen, Denmark; 4 Department of Pathology, Copenhagen University Hospital, Copenhagen, Denmark; 5 Department of Ophthalmology, Aarhus University Hospital, Aarhus, Denmark; 6 The Research Unit for General Practice and Section of General Practice, Department of Public Health, University of Copenhagen, Copenhagen, Denmark

**Keywords:** Eye manifestations, Cancer

## Abstract

**Background:**

Children with brain tumours often suffer from late diagnosis, impacting cure rates and risk of permanent sequelae. Ophthalmological symptoms are common, and we aimed to investigate the frequency, diagnostic interval, and prognostic value of early-onset ophthalmological brain tumour signs.

**Methods:**

The study is based on data from national Danish health registries and medical files from hospitals and private ophthalmologists collected from all children diagnosed with a primary brain tumour in Denmark during 2007–2017.

**Results:**

Among 437 included children, 51.7% (*n *= 226) had ophthalmological tumour signs prior to diagnosis, and 10.8% (*n *= 47) had ophthalmological symptoms as their initial tumour manifestation. The most common ophthalmological signs in total before diagnosis were reduced visual acuity (*n *= 73; 16.7%), diplopia (*n *= 65; 14.9%), abnormal optic nerve (*n *= 59; 13.5%), and strabismus (*n *= 50; 11.4%). The median time from initial symptom onset to diagnosis was 12.6 weeks for all children, 15.9 weeks for those with ophthalmological symptoms as their initial tumour sign (*p *= 0.28), and 12.5 weeks for those with ophthalmological tumour signs at any time before diagnosis (*p *= 0.71). Children with ophthalmological signs before diagnosis had a higher risk of death (HR: 2.11; 95% CI: 1.32–3.39; *p *= 0.002).

**Conclusions:**

Ophthalmological tumour signs are frequent in children with brain tumours, and the diagnostic interval is long regardless of ophthalmological tumour signs being present or not. Taken together with the higher risk of death in the group with ophthalmological tumour signs, this study emphasises the importance of the ophthalmological assessment to ensure timely diagnosis.

## Introduction

Brain tumours are the most frequent solid tumours in children, accounting for 20–25% of childhood neoplasms with an estimated annual incidence of 3–5 per 100.000 children [1]. Furthermore, brain tumours are the leading cause of cancer-related mortality in children and the leading non-accidental cause of childhood death in high-income countries [2–4]. The five-year survival rate has improved to 70–80% in past decades [1, 3]. However, many survivors experience a reduced quality of life due to long-term sequelae such as cognitive, endocrine, and visual impairment [2, 5–10].

Children with brain tumours often suffer from a diagnostic delay [2, 7, 11], which may be associated with an even worse prognosis [7, 8, 12, 13]. An increased interest in earlier detection has emerged in recent years [11, 14, 15]. The time from the onset of the initial tumour sign to the diagnosis is defined as the total diagnostic interval (TDI). Following the introduction of HeadSmart in the UK, a paediatric brain tumour awareness campaign, the median TDI was reduced from 14.4 weeks to 6.7 weeks [14, 16]. Reducing TDI improves the opportunities for earlier and better treatment and, thus, a better prognosis by lowering subsequent morbidity and perhaps even mortality [7, 8, 12, 13, 17].

Initial symptoms often tend to vary and be nonspecific with headache and vomiting being the most common, followed by ophthalmological symptoms [2, 11, 16, 18]. Common eye symptoms, such as reduced visual acuity and strabismus, may not initially be acknowledged as a tumour sign owing to the frequency of these conditions in otherwise healthy children [7, 15, 19, 20].

Enhanced knowledge of ophthalmological tumour signs can potentially reduce the diagnostic interval [7, 21–23] and the aim of this study is consequently to investigate the frequency, diagnostic interval, and prognostic value of ophthalmological tumour signs in Danish children 0–18 years of age with primary brain tumours.

## Materials and methods

This study is a national retrospective study. Using data from the Danish Childhood Cancer Registry (DCCR), we identified all children aged 0–18 years diagnosed with a central nervous system (CNS) tumour in Denmark from 2007 to 2017. DCCR contains information on all children (<18 years) diagnosed with cancer in Denmark [24]. We extracted data from DCCR, including patient ID, the date of diagnosis, tumour location, histopathology, survival status, and date of death (if applicable). We identified a total population of 528 children and obtained hospital files from two years before the diagnosis. The Danish National Health Insurance Service Registry (NHSR) holds information regarding the citizens’ visits to the Danish health care system. Hence, we collected medical files from private ophthalmologists who had examined the children up to two years prior to the diagnosis. The last day of follow-up was the 1^st^ of May 2019.

After reviewing all the medical files, we excluded 91 children, resulting in a total population of 437. Exclusion criteria included tumours with extracerebral origin (*n *= 26), not having a neoplastic tumour (*n *= 17), no connection to the Danish health care system at the time of diagnosis (*n *= 6), lack of sufficient data (*n *= 5), age >18 years old when diagnosed (*n *= 5), and diagnosis before 2007 (*n *= 3). Further, 29 children were excluded because they had a genetic tumour predisposition syndrome that was already known before the diagnosis (neurofibromatosis type 1 *n *= 22; tuberous sclerosis *n *= 3; von Hippel-Lindau syndrome *n *= 2; neurofibromatosis type 2 *n *= 1; Gorlin syndrome *n *= 1). These children are enrolled in a tumour screening program, and the events leading to the diagnosis were, therefore, not comparable to the rest of the group. Children whose tumour predisposition syndromes were not known at the time of the brain tumour diagnosis were included.

Only tumour signs present before the diagnosis were registered. Both subjective complaints and clinical findings were included as tumour signs if they were verified by the clinical examination and/or not found to be caused by another more likely reason. Whether to include a sign as a tumour sign was decided by two medical specialists (one a paediatric ophthalmologist) after a thorough evaluation of the information given in the medical files. If there was doubt about whether to include the sign, another paediatric ophthalmologist was consulted. Parents and the children themselves were the primary observers of initial symptoms. Clinical findings were identified by ophthalmologists, general practitioners (GPs), and paediatricians. Reduced visual acuity included both monocular and binocular impairment. The tests used were Teller and Cardiff for the youngest children, figure charts and Standard Snellen charts with letters for school-age children. Symptoms with onset more than two years before the diagnosis were not recorded unless clearly stated as a tumour sign in the medical files. We registered the initial tumour sign for every child, which was the symptom or clinical finding with the earliest onset. In the analyses, we differentiated between initial signs and signs occurring at any time before the diagnosis (total tumour signs which also included initial tumour signs). The group of children with initial tumour signs being ophthalmological are referred to as the IOS group.

TDI was defined as the interval from the initial tumour sign onset to the diagnosis (defined as the date of tumour identification with CT or MR imaging). Age, tumour grade, and location were subdivided (Table 1), and tumour signs were subcategorised (Table 2).

**Table 1:** Demographics, clinical characteristics, and tumour characteristics of children diagnosed with brain tumours in Denmark from 2007 to 2017 (*n *= 437).

Sex, *n* (%)	
Boys	214 (49.0)
Girls	223 (51.0)
Age at diagnosis, years	
Mean (SD)	8.1 (±4.9)
Median (IQR)	8.0 (8.7)
Age group, *n* (%)	
Under 5 years	144 (33.0)
5–11 years	174 (39.8)
12–18 years	119 (27.2)
Initial tumour sign, *n* (%)	
Ophthalmological	47 (10.8)
General	384 (87.9)
No symptoms	6 (1.4)
Patients with ophthalmological tumour signs before diagnosis, *n* (%)	226 (51.7)
Patients examined by an ophthalmologist before diagnosis, *n* (%)	164 (37.5)
Time from initial tumour sign onset to diagnosis, weeks	
Median (IQR)	12.6 (30.7)
Mean (range)	28.8 (0.0–428.6)
Number of tumour signs at diagnosis, mean (range)	3.9 (0–11)
Tumour grade, *n* (%)	
Low-grade	245 (56.1)
High-grade	137 (31.4)
Unknown or not specified	55 (12.6)
Tumour location, *n* (%)	
Infratentorial	210 (48.1)
Supratentorial	200 (45.8)
Unknown or not specified	27 (6.2)
Deceased at the end of follow-up, *n* (%)	93 (21.3)
Median survival of deceased children, days (range)	380 (0–3467)
5-year survival rate, %	80.1

*SD* standard deviation, *IQR* interquartile range.

**Table 2:** Tumour sign frequency before diagnosis according to diagnostic interval^a^ and demographics^b^ in children with brain tumours (*n *= 437).

Tumour signs	Initial tumour sign		Total tumour signs		Diagnostic interval (days)		Age(years)	Sex			
								Boys		Girls	
	*n*	*%*	*n*	*%*	*Median*	*Mean*	*Median*	*n*	*%*	*n*	*%*
**Ophthalmological signs**											
Reduced visual acuity	14	3.2	73	16.7	46	131	8.8	31	42	42	58
Diplopia	7	1.6	65	14.9	14	25	10.9	26	40	39	60
Abnormal optic nerve	–	–	59	13.5	1	18	8.3	30	51	29	49
Papilledema	–	–	41	9.4	0	1	8.9	20	49	21	51
Optic disc pallor or atrophy	–	–	18	4.1	23.5	56	6.4	10	56	8	44
Strabismus^c^	14	3.2	50	11.4	20.5	89	6.7	21	42	29	58
Esotropia	11	2.5	37	8.5	21	96	4.7	18	49	19	51
Exotropia	2	0.5	7	1.6	30	89	8.2	1	14	6	86
Other or unspecified	1	0.2	7	1.6	12	107	12.5	3	43	4	57
Nystagmus	4	0.9	41	9.4	6	34	7.7	15	37	26	63
Abnormal pupils^c^	–	–	35	8.0	1	14	7.9	16	46	19	54
Anisocoria or dilated pupils	–	–	19	4.3	2	20	7.3	7	37	12	63
Abnormal light response	–	–	23	5.3	1	8	6.6	10	43	13	57
Light sensitivity	–	–	31	7.1	17	78	8.3	18	58	13	42
Visual field defects	–	–	22	5.0	9	31	12.6	6	27	16	73
Hemianopia	–	–	4	0.9	7.5	15	9.4	0	0	4	100
Other or unspecified	–	–	18	4.1	12.5	35	13.0	6	33	12	67
Other motility disturbances	1	0.2	10	2.3	19.5	84	6.5	4	40	6	60
Ptosis	–	–	10	2.3	12	85	7.5	5	50	5	50
Sunset eyes	1	0.2	8	1.8	1.5	50	0.3	4	50	4	50
Colour vision deficiency	–	–	8	1.8	4	13	10.3	3	38	5	63
Proptosis	4	0.9	6	1.4	47.5	160	4.2	1	17	5	83
**Ophthalmological/general signs**											
Abnormal head position	10	2.3	44	10.1	23	69	3.7	23	52	21	48
Cranial nerve palsy^c^	4	0.9	39	8.9	3	22	9.2	19	49	20	51
Sixth nerve palsy	–	–	13	3.0	3	14	8.7	5	38	8	62
Facial nerve palsy	4	0.9	24	5.5	2	25	9.2	12	50	12	50
Other cranial nerve palsies	–	–	4	0.9	3.5	4	8.0	2	50	2	50
**General signs**											
Headache	128	29.3	255	58.4	47	130	9.5	124	49	131	51
Nausea/vomiting	49	11.2	245	56.1	27	76	8.1	114	47	131	53
Change of behaviour	33	7.6	196	44.9	32	86	6.7	92	47	104	53
Ataxia/vertigo/balance problems	38	8.7	170	38.9	26	70	7.9	73	43	97	57
Seizures	52	11.9	89	20.4	54	187	8.6	46	52	43	48
Focal neurological deficits	14	3.2	82	18.8	4.5	48	7.1	36	44	46	56
Endocrine disorders	25	5.7	60	13.7	118	288	8.7	27	45	33	55
Developmental delay	17	3.9	37	8.5	203	472	2.1	22	59	15	41
Increased head circumference	9	2.1	32	7.3	6.5	41	0.9	14	44	18	56
Hearing loss/tinnitus	5	1.1	24	5.4	54.5	127	13.1	13	54	11	46
Respiratory difficulties	2	0.5	3	0.7	106	159	4.6	3	100	0	0
No symptoms^d^	–	–	6	1.4	–	–	12.1	3	50	3	50

^a^Diagnostic interval is defined as the time from the onset of the tumour sign in children presenting the sign to the diagnosis was made.

^b^Information listed in diagnostic interval, age, and sex refers to all the children with the tumour sign before the diagnosis.

^c^Among these tumour signs was a minimum of one child who had two of the subcategorised tumour signs before the diagnosis was made.

^d^Three patients were diagnosed with CTC due to head trauma. One patient was diagnosed with a prenatal ultrasound. One patient was diagnosed due to suspicion of neurofibromatosis type 2 but with no brain tumour signs. One patient was diagnosed with a headache with onset seven years before the diagnosis, but the headache was subsequently not recognised as a tumour symptom and, therefore, not included as such.

Statistical analyses were performed with R (version 4.1.0). Differences in median time to diagnosis were calculated with the nonparametric Mann–Whitney U and Kruskal–Wallis tests (Table 3). The chi-squared test and the t-test were applied for other subgroup comparisons as appropriate.

**Table 3:** Distribution of total diagnostic interval (TDI) according to the occurrence and timing of ophthalmological tumour signs before diagnosis, sex, age, tumour location, tumour grade, and survival status in children with brain tumours.

	Median TDI (weeks)	*p*-value
Initial ophthalmological tumour sign		0.28
Yes	15.9	
No	12.3	
Ophthalmological tumour signs at any time		0.71
Yes	12.5	
No	12.6	
Sex		0.59
Boys	13.1	
Girls	11.8	
Age group		0.02^a^
Under 5 years	8.1	
5–11 years	14.8	
12–18 years	15.6	
Tumour location		0.007^a^
Infratentorial	9.1	
Supratentorial	17.1	
Tumour grade		<0.001^a^
Low-grade	19.3	
High-grade	5.0	
Deceased		<0.001^a^
Yes	6.1	
No	15.0	

^a^Statistical significance.

The relative importance of the various tumour signs for particularly long TDI (three horizons: TDI > 1 month; TDI > 3 months; TDI > 1 year) in each of the three age groups was investigated with dominance analysis. In such analysis, the relative contributions of the tumour signs to the coefficient of determination (R^2^) in a multivariable regression model were calculated by averaging the increase in R^2^ due to an addition of a specific sign to the model, over all possible model configurations [25]. Subsequently, the top 3 signs in each analysis were evaluated in a multivariable logistic regression model to determine the direction of the effect, i.e. odds ratio (OR) < 1 implies shorter TDI when the sign is present and identifies the sign as a helpful diagnostic sign, while OR > 1 implies longer TDI when the sign is present and identifies it as not facilitating the diagnosis within the specified timeframe (Supplementary Table 1).

**Fig. 1:**
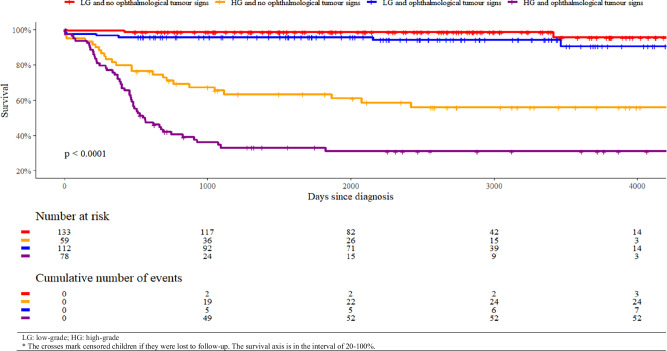
Kaplan–Meier survival curves for children with brain tumours divided according to tumour grade and the occurrence of ophthalmological tumour signs before diagnosis. The figure illustrates worse survival for children with ophthalmological tumour signs compared to children with no ophthalmological tumour signs in both high-grade (HG) and low-grade (LG) tumour groups.

The associations between ophthalmological tumour signs and mortality were evaluated in multivariable Cox regression models. In these models, the occurrence of ophthalmological tumour signs before diagnosis (in three categories: initial sign, subsequent sign, none) was associated with the incidence of (all-cause) death with hazard ratios (HRs) (Table 4). The log-rank test was used to compare Kaplan–Meier survival curves (Fig. 1).

**Table 4:** Cox regression analyses with hazard ratios for death in children with brain tumours related to ophthalmological tumour signs and total diagnostic interval (TDI).

Ophthalmological tumour signs	IR (per 1000 patient-years)	Unadjusted	*p*-value	Adjusted^b^	*p*-value
		HR (95% CI)		HR (95% CI)	
Population					
None	23.4	(ref.)		(ref.)	
Initial	31.2	1.25 (0.57–2.73)	0.57	1.68 (0.74–3.78)	0.21
Subsequent	62.1	2.40 (1.54– 3.75)	<0.001^a^	2.11 (1.32–3.39)	0.002^a^
TDI ≤ 30 days					
None	32.4	(ref.)		(ref.)	
Initial	236.1	5.61 (1.75–18.02)	0.004^a^	4.87 (1.41–16.83)	0.012^a^
Subsequent	109.0	2.94 (1.39–6.22)	0.005^a^	2.80 (1.25–6.29)	0.013^a^
30 < TDI ≤ 90 days					
None	47.4	(ref.)		(ref.)	
Initial	34.5	0.71 (0.20–2.51)	0.59	0.95 (0.23–3.85)	0.94
Subsequent	71.0	1.36 (0.63–2.94)	0.44	1.46 (0.63–3.38)	0.38
90 < TDI ≤ 365 days					
None	9.8	(ref.)		(ref.)	
Initial	9.2	0.85 (0.10–7.24)	0.88	1.07 (0.11–9.99)	0.96
Subsequent	52.0	4.87 (1.76–13.44)	0.002^a^	4.06 (1.11–14.88)	0.035^a^
TDI > 365 days					
None	11.2	(ref.)		(ref.)	
Initial	0.0	–	–	–	–
Subsequent	20.1	1.92 (0.35–10.48)	0.45	1.55 (0.11–21.69)	0.74

*IR* incidence rate, *HR* hazard ratio, *CI* confidence interval, *ref.* reference.

^a^Statistical significance.

^b^Adjusted for age, sex, tumour location, and tumour grade. The analyses for our total population were also adjusted for TDI.

The study was approved by the Danish Patient Safety Authority (3-3013-2800/1) and the Danish Data Protection Agency (VD-2019-84). The study was evaluated by the Regional Research Ethical Committee of the Capital Region, Copenhagen, Denmark, and did not require ethics approval. The study adhered to the Declaration of Helsinki.

## Results

In total, 437 children with a primary brain tumour were included in the study (Table 1). The gender distribution was equal, and the median age at diagnosis was 8.0 years. In our population, 10.8% (*n *= 47) of the children had an ophthalmological tumour sign as their initial sign (IOS group) and 51.7% (*n *= 226) had at least one ophthalmological tumour sign at any time before they were diagnosed (Table 1). In total, 37.5% (*n *= 164) of the children were examined by an ophthalmologist before the diagnosis. Among the 226 children with ophthalmological tumour signs, 53.1% (*n *= 120) were examined by an ophthalmologist before diagnosis (Table 1).

### Ophthalmological tumour signs

The most common initial ophthalmological tumour signs (IOS group) were reduced visual acuity (*n *= 14; 3.2%) and strabismus (*n *= 14; 3.2%) (Table 2). Diplopia (*n *= 7; 1.6%), nystagmus (*n *= 4; 0.9%), and proptosis (*n *= 4; 0.9%) were less common initial ophthalmological signs. Reduced visual acuity (*n *= 73; 16.7%) and strabismus (*n *= 50; 11.4%) were also among the most frequent ophthalmological tumour signs in total prior to diagnosis (Table 2).

### Diagnostic interval and ophthalmological tumour signs

TDI was 12.6 weeks for the 431 children with symptoms. For the IOS group, the median TDI was 15.9 weeks compared with 12.3 weeks for children with non-ophthalmological initial signs (*p *= 0.28) (Table 3). There was no difference in median time to diagnosis for children with ophthalmological tumour signs at any time before the diagnosis and those without (12.5 weeks vs 12.6 weeks; *p *= 0.71).

The ophthalmological signs with the longest median diagnostic interval (time from onset of the sign to diagnosis in children presenting the sign, regardless of when it appeared before diagnosis) were proptosis (47.5 days), reduced visual acuity (46 days) and strabismus (20.5 days) (Table 2). Papilledema was the sign with the shortest diagnostic interval overall (median 0 days; mean 1 day).

### Relative diagnostic importance

Strabismus was one of the initial ophthalmological tumour signs with the highest relative importance (RI) related to the diagnostic interval (Supplementary Table 1). As the initial tumour sign in both the oldest and youngest children, strabismus was a sign that precluded a diagnostic delay of >1 year (OR = 0). In the youngest children, strabismus as an initial tumour sign hindered quick diagnosis, i.e., a TDI < 1 month was not likely (OR = 2.89). Among the oldest children, diplopia had a top-3 highest RI among all tumour signs. For analyses with diagnostic delay >1 month, >3 months, and >1 year, diplopia was an initial tumour sign with ORs in the interval 0–0.20, thereby a sign facilitating faster diagnosis.

### General tumour signs, sex, age and tumour grade, and location

More girls than boys had initial ophthalmological tumour signs (*p *= 0.001) as well as ophthalmological signs at any time before diagnosis (*p *= 0.02) (Supplementary Table 2). Girls had a mean number of total tumour signs before the diagnosis of 4.0, compared with 3.7 for boys (*p *= 0.04). Nearly 90% (*n *= 383) of the children had a minimum of two tumour signs at the time of diagnosis. The most common general tumour signs were headache (*n *= 255; 58.4%), nausea/vomiting (*n *= 245; 56.1%), change of behaviour (*n *= 196; 44.9%), and ataxia/vertigo/balance problems (*n *= 170; 38.9%) (Table 2). The distribution of children with ophthalmological signs at any time before diagnosis according to age groups differed significantly, as proportionally more children had ophthalmological manifestations with increasing age (*p *= 0.03) (Supplementary Table 2). Furthermore, there was a significantly higher distribution of high-grade tumours among the children with ophthalmological signs at any time before diagnosis (*p *= 0.046) (Supplementary Table 2). A significantly higher proportion of high-grade tumours were located infratentorial rather than supratentorial (*p *< 0.001). High-grade tumours and death were highly correlated (*p *< 0.001).

### Survival

In an adjusted multivariable Cox regression analysis for our total population, children with ophthalmological tumour signs before diagnosis (but not as initial signs, i.e. subsequent) had a significantly higher risk of death (HR: 2.11; 95% CI: 1.32–3.39; *p *= 0.002) (Table 4 and Fig. 1). Among children with TDI ≤ 30 days, both initial and subsequent ophthalmological signs were significantly correlated with a higher risk of death (HR: 4.87; 95% CI: 1.41–16.83; *p *= 0.012 and HR: 2.80; 95% CI: 1.25–6.29; *p *= 0.013). In addition, children with a TDI between 91 and 365 days showed a significant association between ophthalmological signs before diagnosis (subsequent) and death (HR: 4.06; 95% CI: 1.11–14.88; *p *= 0.035).

## Discussion

This study provides an overview of ophthalmological and general tumour signs prior to the diagnosis in a nationwide population of 437 children diagnosed with a primary brain tumour during an 11-year period in Denmark. We found a high prevalence of ophthalmological tumour signs before the diagnosis (51.7%), with 10.8% of the population presenting with one of these signs as their initial symptom (IOS group). Reduced visual acuity (16.7%), diplopia (14.9%), abnormal optic nerve (13.5%), and strabismus (11.4%) were the most common ophthalmological signs prior to diagnosis. Having ophthalmological tumour signs was associated with an increased risk of death.

Compared with our 51.7% with ophthalmological tumour signs at any time before diagnosis, both higher and lower frequencies are reported [2, 15, 19, 23, 26]. Nuijts et al. [27] found ophthalmological abnormalities in 79% of children with newly diagnosed brain tumours and also discovered that many children had ophthalmological abnormalities when examined despite not having any ophthalmological complaints. In our study, only 38% of the children were examined by an ophthalmologist before the diagnosis. Of the children with ophthalmological tumour signs, only 53% were seen by an ophthalmologist. Therefore, the prevalence of ophthalmological tumour signs is likely higher than our results show.

Reduced visual acuity and strabismus were among the most frequent ophthalmological tumour signs (16.7% and 11.4%), but also the ophthalmological signs with the most prolonged median diagnostic interval (46 and 20.5 days), which is in agreement with other studies [15, 19]. However, reduced visual acuity and strabismus are also common ophthalmological complaints in the general paediatric population due to less severe conditions such as refractive errors and amblyopia etc. [28–31]. Consequently, common and early-onset ophthalmological tumour signs hold the potential to mimic more frequent and less severe ophthalmological disorders, which might explain the longer diagnostic interval [26]. To distinguish these tumour signs from the common differential diagnoses, factors such as sudden-onset strabismus, pupil abnormalities, and co-existing other general tumour signs should raise suspicion for intracranial pathology [9]. The RI analyses confirmed that the youngest children with strabismus as their initial tumour sign had poor chances of being diagnosed <1 month from the onset of the symptom (OR = 2.89). Only when looking at diagnoses made within one year did the RI analyses demonstrate strabismus as a symptom with low OR (OR = 0), indicating that the diagnosis had a high chance of being established within that timeframe.

Diplopia, the second-most frequent ophthalmological tumour sign (14.9%), had a shorter median time to diagnosis (14 days) than reduced visual acuity and strabismus. The RI analyses for the oldest children with diplopia as their initial tumour sign demonstrated that the symptom was associated with a quick diagnosis, indicating that this symptom is well-recognised as a potential sign of intracranial pathology. Falcone and colleagues [32] reported a higher risk of life-threatening conditions in children with diplopia and associated neurologic signs, which highlights the importance of investigating alarming patterns in children’s presenting symptoms.

Papilledema had a median diagnostic interval of 0 days, which indicates that the children are promptly scanned once the clinical finding is observed. However, how long the papilledema has been present before being diagnosed remains unknown. Papilledema was observed in 9.4% of the children. Nuijts et al. [27] found that papilledema was the most frequent ophthalmological abnormality (52%). The variance in the prevalence of papilledema in the studies may be explained by the fact that Nuijts et al. [27] examined all children after a standardised protocol performed by ophthalmologists, whereas not all children in our study underwent ophthalmological assessments. Papilledema is an important and well-known sign of increased intracranial pressure that can lead to permanent vision loss, making early detection and intervention crucial [22]. However, it requires clinical assessment to be recognised and taken together, this illustrates the importance of timely ophthalmological evaluation.

In infants and young children with open fontanelles, macrocephaly and sunset eyes can be signs of raised intracranial pressure [33, 34], and these signs also had short median diagnostic intervals (6.5 days and 1.5 days). Other tumour signs with short median diagnostic intervals included pupil abnormalities (1 day), cranial nerve palsies (3 days), nystagmus (6 days), and visual field defects (9 days). These signs share the characteristic that they have a higher predictive value for raised intracranial pressure and intracranial space-occupying lesions [7, 9, 15, 22, 35].

The occurrence of ophthalmological tumour signs at any time before diagnosis differed significantly in the age groups, with more children having ophthalmological signs in the older groups (5–11 and 12–18 years compared with <5 years). This association might be due to the ability to describe more complex symptoms, better cooperation, and biological development [2, 9, 11, 21]. The youngest children and particularly pre-verbal children cannot describe their symptoms in the same manner as older children and the tumour signs observed are only those that the parents may observe and the results of the clinical examination.

Girls had significantly more tumour signs than boys. Other studies have shown a similar trend in other CNS-related diseases in adolescents and adults [36–38]. However, little is known about the differences in symptomatology in children according to gender. In our study, girls also constituted a significantly higher proportion of the children with ophthalmological signs.

Overall, the median TDI was 12.6 weeks, ranking poorly compared with other studies with TDI varying from a few weeks to 2–3 months [11, 13, 19, 26, 39]. The IOS group had a longer TDI than children with other initial signs (15.9 vs 12.3 weeks), but the difference was not significant. In our study, we did not differentiate between patient delay (symptom onset to first medical consultation) and the system interval (first health care contact to diagnosis) [14]. Therefore, the longer TDI in our IOS group may be the result of a lack of awareness in both the general population and among health care professionals. Diagnostic delay may, however, be influenced by a number of other factors including socioeconomic aspects [40]. In Denmark, patients typically consult a GP before referral to an ophthalmologist or paediatric specialist, but they can also access private ophthalmologists directly, with all health care services being free of cost and covering the entire population. Despite this, socioeconomic factors play an important role in how the system is used in Denmark [41]. A campaign similar to HeadSmart (“Hjernetegn”) has been introduced in Denmark to guide and inform health care professionals how to act if specific symptoms occur, which tumour signs to be aware of, and when the child should be referred [42]. Hopefully, this initiative will help reduce system delay.

We found a higher distribution of high-grade tumours in children with ophthalmological tumour signs at any time prior to diagnosis than those without (41.1% vs 30.7%; *p *= 0.046). It is well-established that high-grade tumours are associated with both death and short TDI [11, 14, 15, 43, 44]. High-grade tumours grow more rapidly and aggressively, thus giving a higher risk of infiltration and compression of visual pathway structures and involvement of more anatomical sites [11, 34]. However, even when adjusting for tumour grade, we found an association between ophthalmological tumour signs before diagnosis and death for both our total population (HR: 2.11; 95% CI: 1.32–3.39; *p *= 0.002) and the subgroup of patients with short TDI (HR: 2.80; 95% CI: 1.25–6.29; *p *= 0.013). Specifically for the children with short TDI, we found that the IOS group had a higher risk of death (HR: 4.87; 95% CI: 1.41–16.83; *p *= 0.012), emphasising the importance of awareness of early ophthalmological tumour signs. In addition, children with a TDI between 91 and 365 days combined with ophthalmological tumour signs before diagnosis had a significantly higher risk of death in adjusted Cox regression analyses. Some studies have demonstrated an association between a TDI of 3–6 months and death [12, 43], suggesting that tumours diagnosed within an intermediate diagnostic interval could be of specific histopathologic subtypes or tumour locations, causing this link between death, ophthalmological tumour signs, and diagnostic interval. This association between ophthalmological tumour signs and an increased risk of death may be because children with ophthalmological tumour signs have tumours at a more advanced stage, where there is already growth into regions not yet affected in children without eye symptoms at the time of diagnosis.

Although short TDI is paradoxically associated with a poor prognosis, studies have also shown that reducing the diagnostic interval may improve survival and the long-term sequelae [7, 8, 12, 13, 17]. Earlier tumour detection allows initiating treatment at a less progressive stage and improves treatment options [40, 45, 46]. For a GP, a paediatrician, or an ophthalmologist, information about how the child develops, any behavioural changes, visual disturbances, and other associated potential tumour signs should be routine and will often bring crucial knowledge to the health care professional. Clinical assessment for other tumour signs that might otherwise have been left unnoticed and awareness of the number and combination of potential tumour signs are essential, and children should be referred for further evaluation at the slightest doubt to ensure faster diagnosis.

### Strengths and limitations

Major strengths were the nationwide collection of high-quality data, enabling us to collect medical information from hospitals and private ophthalmologists of most children. DCCR data regarding diagnosis date, tumour grade, and location were validated in the medical files. Owing to the availability of detailed data on tumour location and previous eye history, we could distinguish tumour signs from other ophthalmological issues in most cases.

However, our methods imply a risk of including symptoms or clinical findings not caused by a brain tumour. The retrospective study design implies that only reported information from our collected files from hospitals and private ophthalmologists was available. In addition, the calculations of diagnostic intervals may be affected by recall bias. The diagnostic interval did not distinguish between patient interval and system interval and did not include socioeconomic background. This study does not analyse how specific tumour sign patterns correlate with survival status, tumour grade, or location.

## Conclusion

Children with brain tumours in Denmark have a prolonged diagnostic interval, but this does not differ in children with or without ophthalmological tumour signs. Ophthalmological tumour signs are frequent and associated with a worse prognosis, emphasising the importance of timely standardised ophthalmological referral and assessment performed by ophthalmologists to reduce the diagnostic interval and improve the prognosis for children with brain tumours.

## Summary

### What was known before

- Children with brain tumours often suffer from late diagnosis, impacting cure rates and risk of permanent sequelae.
- Ophthalmological symptoms are common.

### What this study adds

- This study confirms that time to diagnosis is prolonged for children with brain tumours.
- Ophthalmological symptoms are common and observed in more than 50% of the children included in this study.
- Children with ophthalmological tumour signs have a higher mortality compared to those without.
- Our findings emphasise the importance of early ophthalmological assessments in ensuring timely diagnosis of children with brain tumours.

## Supplementary information

Supplementary Table 1

Supplementary Table 2

## Data Availability

The majority of data generated or analysed during this study is available in this published article (and its supplementary information files). Further detailed data are not publicly available owing to individual privacy of the children included in the study.
