# Supplementary material for: Early ophthalmological tumour signs and diagnostic interval in children with brain tumours
Source: Eye (Lond). 2025 May 17;39(11):2245–52. doi: 10.1038/s41433-025-03837-8 (PMC12274472; doi:10.1038/s41433-025-03837-8)
Supplement: Supplementary file 1 — Supplementary Table 1 [file 41433_2025_3837_MOESM1_ESM.pdf]

Supplementary Table 1: The relative importance of ophthalmological and general tumour signs, appearing as initial or subsequent signs before diagnosis, for diagnostic delays larger than 1 month, 3 months and 1 year. For the top-3 signs, and additionally the top-3 ophthalmological signs, the odds ratio for long diagnostic delay is estimated; OR < 1 indicates a symptom associated with shorter diagnostic delay, OR > 1 indicates a symptom associated with longer diagnostic delay.

		Age under 5 years									Age 5-11 years									Age 12-18 years								
		Diagnostic delay > 1 month			Diagnostic delay > 3 months			Diagnostic delay > 1 year			Diagnostic delay > 1 month			Diagnostic delay > 3 months			Diagnostic delay > 1 year			Diagnostic delay > 1 month			Diagnostic delay > 3 months			Diagnostic delay > 1 year		
		RI	98/143	OR (95% CI)	RI	58/143	OR (95% CI)	RI	20/143	OR (95% CI)	RI	131/172	OR (95% CI)	RI	90/172	OR (95% CI)	RI	27/172	OR (95% CI)	RI	87/116	OR (95% CI)	RI	64/116	OR (95% CI)	RI	20/116	OR (95% CI)
**Ophthalmological tumour signs**																												
Reduced visual acuity	Initial	0,7%	2/3		4,0%	0/3	0	2,1%	0/3		4,2%	10/11		1,5%	9/11		4,0%	4/11		3,1%	7/7		2,5%	4/7		3,1%	1/7	
	Subsequent		9/10			6/10	0.94 (0.17-5.14)		2/10			18/20			12/20			5/20			18/22			15/22			6/22	
	No		87/130			52/130	ref.		18/130			103/141			69/141			18/141			62/87			45/87			13/87	
Diplopia	Initial	0,9%	0/0		1,4%	0/0		0,1%	0/0		8,1%	3/7	0.22 (0.04-1.35)	2,0%	2/7		3,6%	0/7		10,4%	2/5	0.03 (0.00-0.86) *	10,6%	1/5	0.20 (0.01-2.87)	14,8%	0/5	0
	Subsequent		2/3			1/3			0/3			18/27	0.53 (0.18-1.57)		9/27			3/27			16/23	0.38 (0.11-1.38)		9/23	0.36 (0.11-1.14)		0/23	0
	No		96/140			57/140			20/140			110/138	ref.		79/138			24/138			69/88	ref.		54/88	ref.		20/88	ref.
Abnormal optic nerve	Initial	1,9%	1/2		1,8%	0/2		0,9%	0/2		4,3%	0/0	-	0,2%	0/0		0,7%	0/0		8,3%	0/0	-	11,6%	0/0	-	10,1%	0/0	-
	Subsequent		13/15			6/15			3/15			21/25	3.54 (0.90-13.88)		12/25			3/25			15/17	3.73 (0.61-22.84)		12/17	3.38 (0.81-14.03)		5/17	4.56 (1.03-20.21) *
	No		84/126			52/126			17/126			110/147	ref.		78/147			24/147			72/99	ref.		52/99	ref.		15/99	ref.
Strabismus	Initial	6,6%	9/10	2.89 (0.21-40.74)	1,3%	5/10		6,4%	0/10	0	2,2%	2/2		0,5%	1/2		5,2%	1/2	inf.	5,6%	3/3		8,1%	3/3		9,7%	0/3	0
	Subsequent		7/9	5.73 (0.56-58.61)		4/9			1/9	4.05 (0.24-67.42)		13/18			9/18			4/18	4.31 (0.84-22.03)		5/8			3/8			0/8	0
	No		82/124	ref.		49/124			19/124	ref.		116/152			80/152			22/152	ref.		79/105			58/105			20/105	ref.
Nystagmus	Initial	5,8%	4/5	0.29 (0.02-4.06)	3,7%	1/5		3,5%	0/5		0,6%	0/0		2,0%	0/0		1,2%	0/0		3,5%	0/0		0,6%	0/0		1,2%	0/0	
	Subsequent		8/8	inf.		4/8			2/8			12/18			10/18			3/18			10/10			6/10			3/10	
	No		86/130	ref.		53/130			18/130			119/154			80/154			24/154			77/106			58/106			17/106	
Abnormal pupils	Initial	1,3%	1/2		0,6%	0/2		1,7%	0/2		2,9%	0/0		0,5%	0/0		1,3%	0/0		1,1%	0/0		1,7%	0/0		2,9%	0/0	
	Subsequent		6/8			4/8			1/8			14/20			11/20			4/20			4/5			2/5			0/5	
	No		91/133			54/133			19/133			117/152			79/152			23/152			83/111			62/111			20/111	
Light sensitivity	Initial	3,2%	0/0		2,0%	0/0		0,3%	0/0		2,6%	1/2		4,1%	0/2	0	1,4%	0/2		4,4%	4/7		1,2%	3/7		3,3%	1/7	
	Subsequent		0/2			0/2			0/2			10/16			7/16	1.15 (0.30-4.48)		3/16			4/4			3/4			0/4	
	No		98/141			58/141			20/141			120/154			83/154	ref.		24/154			79/105			58/105			19/105	
Visual field defects	Initial	5,2%	0/1		3,7%	0/1		5,4%	0/1	0	2,6%	0/0		3,6%	0/0		6,2%	0/0	-	1,6%	0/0		1,7%	0/0		1,6%	0/0	
	Subsequent		1/1			1/1			1/1	inf.		7/7			6/7			3/7	23.15 (1.73-309.81) *		12/13			10/13			3/13	
	No		97/141			57/141			19/141	ref.		124/165			84/165			24/165	ref.		75/103			54/103			17/103	
Other motility disturbances	Initial	0,3%	0/0		0,7%	0/0		0,7%	0/0		1,1%	1/1		3,8%	0/1	0	4,3%	0/1		0,8%	0/0		1,3%	0/0		0,5%	0/0	
	Subsequent		3/4			2/4			0/4			2/3			2/3	6.44 (0.29-141.17)		1/3			2/2			1/2			0/2	
	No		95/139			56/139			20/139			128/168			88/168	ref.		26/168			85/114			63/114			20/114	
Ptosis	Initial	3,4%	1/1		0,8%	0/1		0,3%	0/1		6,1%	0/1	0	3,4%	0/1		2,5%	0/1		5,2%	0/0		2,0%	0/0		0,4%	0/0	
	Subsequent		1/2			1/2			1/2			3/4	1.74 (0.11-27.74)		2/4			1/4			0/2			0/2			0/2	
	No		96/140			57/140			19/140			128/167	ref.		88/167			26/167			87/114			64/114			20/114	
Sunset eyes	Initial	6,4%	1/1	inf.	2,2%	1/1		2,8%	0/1		0,0%	0/0		0,0%	0/0		0,0%	0/0		0,0%	0/0		0,0%	0/0		0,0%	0/0	
	Subsequent		3/7	0.07 (0.00-1.00)		2/7			0/7			0/0			0/0			0/0			0/0			0/0			0/0	
	No		94/135	ref.		55/135			20/135			131/172			90/172			27/172			87/116			64/116			20/116	
Colour vision deficiency	Initial	0,0%	0/0		0,0%	0/0		0,0%	0/0		0,7%	0/0		1,7%	0/0		0,4%	0/0		0,6%	0/0		1,4%	0/0		4,5%	0/0	
	Subsequent		0/0			0/0			0/0			4/4			4/4			2/4			3/4			2/4			0/4	
	No		98/143			58/143			20/143			127/168			86/168			25/168			84/112			62/112			20/112	
Proptosis	Initial	0,7%	3/3		1,6%	1/3		5,8%	0/3	0	0,3%	1/1		0,6%	1/1		4,4%	1/1	inf.	3,3%	0/0		0,0%	0/0		0,0%	0/0	
	Subsequent		1/1			1/1			1/1	inf.		0/0			0/0			0/0	-		1/1			0/1			0/1	
	No		94/139			56/139			19/139	ref.		130/171			89/171			26/171	ref.		86/115			64/115			20/115	
**Ophthalmological/general tumour signs**																												
Abnormal head position	Initial	4,3%	7/10		8,2%	5/10	0.42 (0.06-2.81)	3,0%	4/10		4,1%	0/0		1,3%	0/0		0,6%	0/0		10,2%	1/1	inf.	10,2%	0/1	0	0,5%	0/1	
	Subsequent		10/17			4/17	0.06 (0.01-0.51) **		1/17			12/14			7/14			2/14			2/2	inf.		0/2	0		0/2	
	No		81/116			49/116	ref.		15/116			119/158			83/158			25/158			84/113	ref.		64/113	ref.		20/113	
Cranial nerve palsy	Initial	0,6%	1/1		5,9%	1/1	inf.	3,5%	0/1		1,6%	3/4		10,3%	0/4	0	0,7%	0/4		3,0%	0/1		7,7%	0/1		3,7%	0/1	
	Subsequent		3/7			3/7	10.04 (1.31-77.14) *		1/7			14/18			7/18	0.85 (0.23-3.10)		1/18			5/8			2/8			1/8	
	No		94/135			54/135	ref.		19/135			144/150			83/150	ref.		26/150			82/107			62/107			19/107	
**General tumour signs**																												
Headache	Initial	2,3%	11/20		10,3%	3/20	0.45 (0.09-2.33)	7,2%	0/20	0	3,1%	56/78		5,6%	33/78		3,9%	9/78		11,2%	42/58	1.56 (0.42-5.86)	14,0%	31/58	2.68 (0.82-8.79)	7,5%	9/58	
	Subsequent		17/22			12/22	5.78 (1.57-21.36) **		4/22	0.65 (0.08-5.53)		33/45			22/45			7/45			29/32	6.63 (1.02-43.13) *		23/32	7.94 (1.80-34.91) **		9/32	
	No		70/101			43/101	ref.		16/101	ref.		42/49			35/49			11/49			16/26	ref.		10/26	ref.		2/26	
Nausea/vomiting	Initial	7,5%	24/35		7,7%	6/35		2,9%	2/35		11,3%	31/51		15,8%	16/51	0.16 (0.05-0.45) **	1,1%	4/51		1,5%	19/26		0,8%	13/26		7,7%	2/26	
	Subsequent		32/40			20/40			3/40			43/55			26/55	0.24 (0.09-0.65) **		7/55			29/38			23/38			10/38	
	No		52/68			32/68			15/68			57/66			48/66	ref.		16/66			39/52			28/52			8/52	
Change of behaviour	Initial	11,3%	14/32	0.22 (0.06-0.74) *	5,4%	7/32		3,9%	2/32		12,2%	19/28	0.76 (0.23-2.51)	16,2%	8/28	0.22 (0.07-0.71) *	11,9%	1/28	0.05 (0.00-0.78) *	1,1%	7/13		1,1%	6/13		3,0%	2/13	
	Subsequent		34/45	1.11 (0.32-3.79)		20/45			6/45			42/49	2.81 (0.96-8.24)		27/49	2.01 (0.74-5.41)		9/49	0.73 (0.18-2.88)		23/29			18/29			3/29	
	No		50/66	ref.		31/66			12/66			70/95	ref.		55/95	ref.		17/95	ref.		57/74			40/74			15/74	
Ataxia/vertigo/balance problems	Initial	10,1%	19/26	4.52 (1.07-19.08) *	1,9%	9/26		3,6%	1/26		1,0%	13/17		1,4%	8/17		1,0%	1/17		4,8%	13/17		2,2%	9/17		1,4%	2/17	
	Subsequent		24/31	5.80 (1.31-25.63) *		14/31			5/31			32/44			19/44			4/44			28/35			21/35			7/35	

	No		55/86	ref.		35/86			14/86			86/111			63/111		22/111			46/64		34/64		11/64
Seizures	Initial	3,1%	10/14		2,6%	7/14		6,1%	1/14		0,8%	15/17		3,4%	12/17	3,7%	2/17		0,7%	14/18	2,6%	9/18	5,8%	5/18
	Subsequent		10/19			6/19			3/19			9/11			5/11		2/11			7/10		6/10		1/10
	No		78/110			45/110			16/110			107/144			73/144		23/144			66/88		49/88		14/88
Focal neurological deficits	Initial	3,3%	3/4		6,1%	2/4		3,8%	0/4		11,3%	4/7	0.43 (0.08-2.43)	7,3%	3/7	6,1%	0/7		7,2%	1/3	3,6%	1/3	8,6%	0/3
	Subsequent		17/27			16/27			8/27			16/28	0.38 (0.13-1.10)		10/28		1/28			8/13		8/13		4/13
	No		78/112			40/112			12/112			111/137	ref.		77/137		26/137			78/100		55/100		16/100
Endocrine disorders	Initial	4,6%	3/4		1,9%	1/4		6,8%	0/4	0	14,0%	16/16	inf.	7,9%	14/16	12,3%	8/16	25.06 (3.24-193.70) **	4,0%	10/11	7,1%	9/11	0,8%	2/11
	Subsequent		11/12			7/12			4/12	6.43 (0.71-58.10)		11/11	inf.		9/11		3/11	1.09 (0.12-9.64)		6/6		5/6		1/6
	No		84/127			50/127			16/127	ref.		104/145	ref.		67/145		16/145	ref.		71/99		50/99		17/99
Developmental delay	Initial	14,3%	17/17	inf.	24,9%	13/17	15.34 (3.33-70.59) **	26,7%	9/17	21.65 (3.92-119.69) **	1,0%	2/2		4,0%	2/2	18,2%	2/2	inf.	2,2%	1/1	3,8%	1/1	0,2%	0/1
	Subsequent		13/15	2.88 (0.44-18.91)		12/15	59.27 (6.84-513.50) **		6/15	15.49 (1.85-129.62) *		2/2			2/2		2/2	inf.		0/0		0/0		0/0
	No		68/111	ref.		33/111	ref.		5/111	ref.		127/168			86/168		23/168	ref.		86/115		63/115		20/115
Increased head circumference	Initial	2,1%	4/9		1,7%	2/9		2,6%	0/9		1,7%	0/0		0,1%	0/0	0,0%	0/0		0,5%	1/1	3,1%	0/1	0,8%	0/1
	Subsequent		16/21			13/21			5/21			1/1			0/1		0/1			0/0		0/0		0/0
	No		78/113			43/113			15/113			130/171			90/171		27/171			86/115		64/115		20/115
Hearing loss/tinnitus	Initial	0,0%	0/0		0,0%	0/0		0,0%	0/0		2,2%	2/3		3,0%	2/3	5,3%	0/3		5,9%	5/5	1,3%	4/5	8,2%	4/5
	Subsequent		0/0			0/0			0/0			5/6			2/6		0/6			9/10		6/10		2/10
	No		98/143			58/143			20/143			124/163			86/163		27/163			73/101		54/101		14/101

RI: relative importance; OR: odds ratio; CI: confidence interval; ref.: reference; inf.: infinitive; *: significant at 5% level; **: significant at 1% level
