# Supplementary material for: Early ophthalmological tumour signs and diagnostic interval in children with brain tumours
Source: Eye (Lond). 2025 May 17;39(11):2245–52. doi: 10.1038/s41433-025-03837-8 (PMC12274472; doi:10.1038/s41433-025-03837-8)
Supplement: Supplementary file 2 — Supplementary Table 2 [file 41433_2025_3837_MOESM2_ESM.pdf]

Supplementary Table 2: Distribution of sex, age, tumour location, and tumour grade according to the timing of the ophthalmological tumour signs prior to diagnosis in children with brain tumours

	Initial ophthalmological tumour sign			Ophthalmological tumour signs at any time		
	Yes	No	p-value	Yes	No	p-value
	n (%)	n (%)		n (%)	n (%)	
**Sex**			0.001 *			0.02 *
Boys	**12** (25.5)	**202** (51.8)		**98** (43.4)	**116** (55.0)	
Girls	**35** (74.5)	**188** (48.2)		**128** (56.6)	**95** (45.0)	
**Age group**			0.42			0.03 *
Under 5 years	**19** (40.4)	**125** (32.1)		**62** (27.4)	**82** (38.9)	
5-11 years	**15** (31.9)	**159** (40.8)		**94** (41.6)	**80** (37.9)	
12-18 years	**13** (27.7)	**106** (27.2)		**70** (31.0)	**49** (23.2)	
**Tumour location**			0.14			0.57
Infratentorial	**17** (39.5)	**193** (52.6)		**113** (52.8)	**97** (49.5)	
Supratentorial	**26** (60.5)	**174** (47.4)		**101** (47.2)	**99** (50.5)	
**Tumour grade**			0.38			0.046 *
Low-grade	**26** (72.2)	**219** (63.3)		**112** (58.9)	**133** (69.3)	
High-grade	**10** (27.8)	**127** (36.7)		**78** (41.1)	**59** (30.7)	

* Statistical significance
